# Primary gastric synovial sarcoma resected by laparoscopic endoscopic cooperative surgery of the stomach: a case report

**DOI:** 10.1186/s40792-021-01310-8

**Published:** 2021-10-20

**Authors:** Ryosuke Shibata, Marina Morishita, Nobuhiko Koreeda, Yousuke Hirano, Hiroki Kaida, Toshihiro Ohmiya, Shugo Uwatoko, Makoto Kawamoto, Akira Komono, Ryohei Sakamoto, Yoshihiro Miyasaka, Daijiro Higashi, Hiroshi Tanabe, Satoshi Nimura, Masato Watanabe

**Affiliations:** 1 Department of Surgery, Fukuoka University Chikushi Hospital, 1-1-1 Zokumyoin, Chikushinoshi, Fukuoka 818-8502 Japan; 2 Department of Pathology, Fukuoka University Chikushi Hospital, 1-1-1 Zokumyoin, Chikushinoshi, Fukuoka 818-8502 Japan

**Keywords:** Synovial sarcoma, Stomach, Laparoscopic endoscopic cooperative surgery

## Abstract

**Background:**

Primary gastric synovial sarcoma is extremely rare, only 44 cases have been reported so far, and there have been no reports of laparoscopic endoscopic cooperative surgery for this condition.

**Case presentation:**

A 45-year-old male patient presented with gastric pain. Esophagogastroduodenoscopy was performed that led to the identification of an 8-mm submucosal tumor in the anterior wall of the antrum, and a kit-negative gastrointestinal stromal tumor was suspected following biopsy. On endoscopic ultrasonography, the boundary of the tumor, mainly composed of the second layer, was depicted as a slightly unclear low-echo region, and a pointless no echo region was scattered inside. A boring biopsy revealed synovial sarcoma. Positron emission tomography did not reveal fluorodeoxyglucose (^18^F-FDG) accumulation in the stomach or other organs. Thus, the patient was diagnosed with a primary gastric synovial sarcoma, and laparoscopic endoscopic cooperative surgery was performed. The tumor of the antrum could not be confirmed laparoscopically from the serosa, and under intraoperative endoscopy, it had delle on the mucosal surface, which was removed by a method that does not involve releasing the gastric wall. Immunohistochemistry showed that the spindle cells were positive for EMA, BCL-2 protein, TLE-1, and SS18-SSX fusion-specific antibodies but negative for KIT and DOG-1. The final pathological diagnosis was synovial sarcoma of the stomach. The postoperative course was good, and the patient was discharged from the hospital on the 11th postoperative day.

**Conclusion:**

Resection with laparoscopic endoscopic cooperative surgery (LECS), which has not been reported before, was effective for small synovial sarcomas that could not be confirmed laparoscopically. With the combination of laparoscopic and endoscopic approaches to neoplasia with a non-exposure technique (CLEAN-NET) procedure, it was possible to excise the tumor with the minimum excision range of the gastric serosa without opening the stomach.

**Supplementary Information:**

The online version contains supplementary material available at 10.1186/s40792-021-01310-8.

## Background

Synovial sarcoma, a malignant soft tissue tumor that occasionally occurs in the limbs of young people, accounts for approximately 10% of all malignant soft tissue tumors [1, 2]. However, it is possible that synovial tissue is not always the tissue of origin of the tumor, and synovial sarcoma can occur throughout the body. Primary synovial sarcoma of the stomach has been reported in only a few dozen cases thus far. Therefore, there is no established protocol for the standard treatment or surgical technique for gastric synovial sarcoma. Here, we report a case of primary gastric synovial sarcoma treated for the first time with LECS to minimize the extent of resection and to preserve function as much as possible.

## Case presentation

**Fig. 1:**
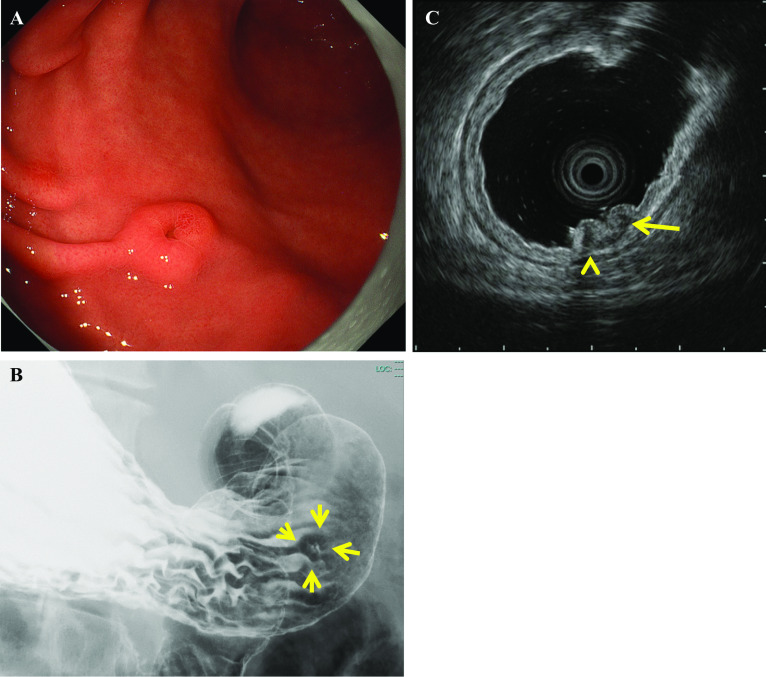
**A** Esophagogastroduodenoscopy findings during the preoperative examination for gastric submucosal tumors. An elevated lesion, 8 mm in size, with depression in the center, covered with normal mucosa was observed in the anterior wall of the antrum. **B** Upper gastrointestinal series findings. A raised lesion with a central depression, 8 mm in size, was found in the greater curvature of the gastric body (arrow). **C** Endoscopic ultrasonography findings. A 10-mm tumor was found in the second layer (arrow). Because of the thinning of the third layer, submucosal invasion could not be completely ruled out (arrow head)

**Fig. 2:**
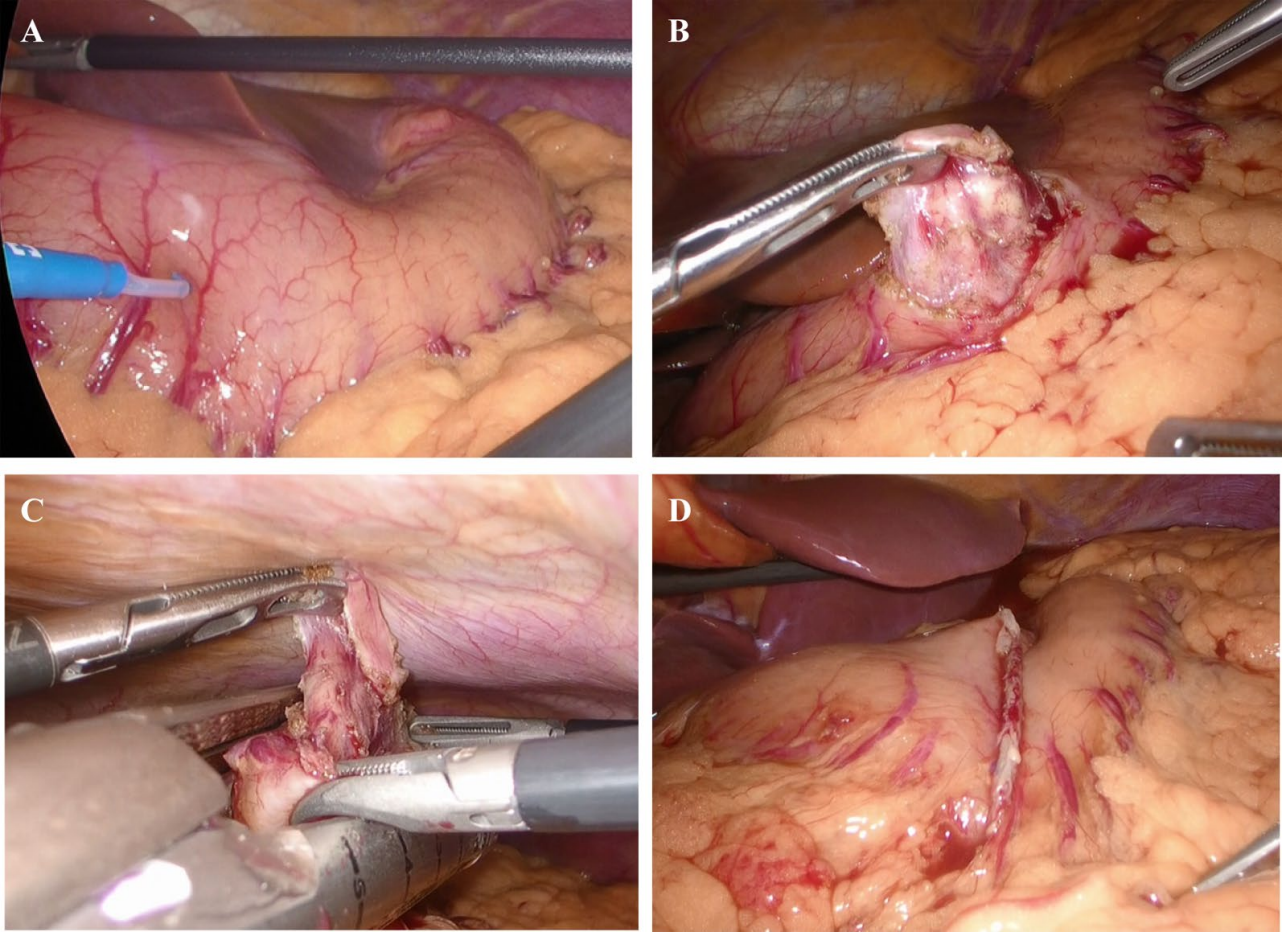
Intraoperative imaging by laparoscopy. **A** The tumor could not be identified from the gastric serosa. **B** The seromuscular layer and submucosal layers were incised completely along the tumor, and the tumor covered with mucosa was towed outside the gastric wall. **C** The whole layer was excised by linear stapler. **D** After tumor resection

**Fig. 3:**
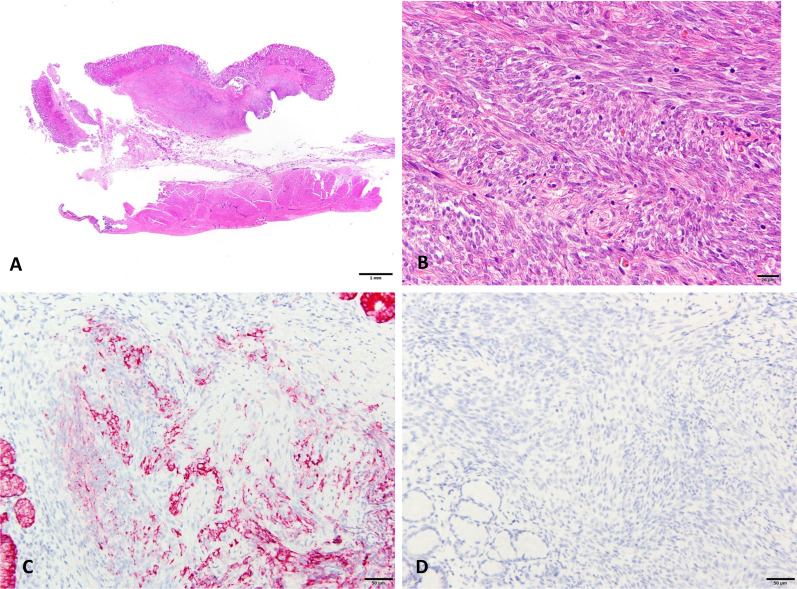
Pathological findings of the tumor. **A** Whole-mount view of the cut section of the tumor. The tumor was mainly located in the submucosal tissue (hematoxylin and eosin [H&E] stain). **B** The tumor is composed of a highly cellular fascicle of short spindle cells. (H&E stain, original magnification, ×400). **C** Immunohistochemical stain using an EMA antibody showed cytoplasmic positivity. (Original magnification, ×200). **D** Lack of DOG-1 expression in spindle cells. (Original magnification, ×200)

The patient was a 45-year-old man complaining of epigastric pain. Esophagogastroduodenoscopy (EGD) was performed by a referral physician, and a submucosal tumor (SMT) was noted on the anterior wall of the stomach antrum; he was, thus, referred to our institute. There were no special notes in the medical history or family history, and the abdomen was flat and soft. Blood biochemical testing was largely normal. During EGD and upper gastrointestinal series, a mass about 8 mm in size covered with normal mucosa with a depression in the center was noted at the antrum of the stomach (Fig. 1A–B). Endoscopic ultrasonography revealed a hypoechoic region of approximately 10 mm in size, mainly in the second layer and thinning of the third layer of the submucosa. Therefore, deep invasion of the submucosa could not be completely ruled out (Fig. 1C). A boring biopsy specimen showed proliferation of uniform atypical short spindle or oval cells. Immunohistochemically, the tumor cells were positive for EMA, BCL-2 protein, TLE-1, and SS18-SSX fusion-specific antibodies but negative for KIT and DOG-1. Molecular genetic analysis by fluorescence in situ hybridization (FISH) using an SS18 break-apart probe revealed SS18 rearrangement. These findings are consistent with those of synovial sarcoma. Abdominal contrast-enhanced computed tomography was not able to identify the lesion, and no lymph node swelling or metastases to other organs was observed. Positron emission tomography also revealed no accumulation of ^18^F-FDG in the gastric lesion or other organs. Primary synovial sarcoma of the stomach was diagnosed, and laparoscopic endoscopic cooperative surgery was chosen as the treatment modality to perform a full thickness resection of the gastric wall for complete resection of the tumor. The operation was performed via five ports. As the gastric lesion could not be confirmed from the serosa surface by laparoscopy (Fig. 2A), and because delle was suspected on endoscopy, we decided to remove it with CLEAN-NET to prevent dissemination (Additional file 1). Enoscopically, glycerol was injected locally under the mucosa around the tumor, causing the mucosa to float. The whole tumor circumference was marked on the serosa laparoscopically, and the endoscope was used as a guide around the tumor. The seromuscular layer and the submucosal layer were completely cut, using the mark as a guide (Fig. 2B). The tumor covered in the mucosa was towed outside the gastric wall, and whole-layer resection was performed using a 60-mm linear stapler to confirm that the tumor was not sandwiched (Fig. 2C–D). The operating time was 116 min, and 5 mL of blood was lost. Histopathologically, the single tumor was composed of short spindle cells of the submucosal tissue and lamina propria mucosae (Fig. 3A–B). Immunohistochemically, the tumor cells showed the same phenotype as that of the biopsied specimen (Fig. 3C–D). The tumor was diagnosed as a synovial sarcoma. The surgically resected margins were tumor-free. During the operation, another SMT < 2 cm was discovered in the lesser curvature of the stomach body by accident and was removed using the classical LECS method. The 116 min of operating time and blood loss of 5 ml were inclusive of this procedure. Histopathological examination revealed the tumor to be a low-risk gastrointestinal stromal tumor. The patient’s postoperative course was good, and he was discharged from the hospital on the 11th postoperative day without any complication. No recurrence was observed 5 months postoperatively.

## Discussion

Synovial sarcoma accounts for approximately 10% of all malignant soft tissue tumors and occurs in the extremities, but has also been reported to occur in various other regions, such as the head and neck, lungs, mediastinum, abdomen, and retroperitoneum [1, 2]. Features of synovial sarcoma include chromosomal translocation (X;18) (p11;q11), found in 95% or more cases genetically, regardless of the development site or histology. This transdermal translocation fuses the *SXY* gene on chromosome 18 with the *SSX1* or *SSX2* gene on the X chromosome to form the *SYT-SSX* chimeric gene. The specific mechanism of action of this gene product in the development of synovial sarcoma is still unknown, but it is thought to be involved in regulating transcriptional activity [3–5]. Recently, TLE1, a Groucho/Transducin-like enhancer of split TLE families, was found to be a diagnostic marker for synovial sarcoma [6]. TLE1 acts as a transcription factor corepressor in various pathways, including the Wnt/β-catenin pathway, suggesting that increased expression of TLE1 may cause abnormalities in downstream gene expression [7]. In this case, TLE-1 and SS18-SSX were positive on immunohistochemical analysis, and synovial sarcoma was diagnosed by biomolecular assessment and genetic identification of SS18 by FISH. As of 2021, there were only 44 (22 men and 22 women) reported cases of primary synovial sarcoma in the stomach [8–31] (Table 1), and the median age of the affected patients was 45 years. Tumors were often locally located in the body and fundus of the stomach, with a median size of 5.46 cm; many also had ulcers. Recently, reports of this tumor type have been increasing [24, 27–30], which may be a result of improved understanding of this tumor type and advances in diagnostic ability.

**Table 1:** Clinical characteristics of the gastric synovial sarcoma (A review of literature)

Number	Sex	Age	Tumor size (in mm)	Treatment	Outcome	Year of publication, reference number
1	M	47	52	Gastrectomy and partial esophagectomy	AND	2000, [8]
2	F	55	160	Hemigastrectomy	DD	2000, [8]
3	M	42	115	Tumorectomy and chemotherapy	DD	2007, [9]
4	F	67	8	Partial gastrectomy	AND	2008, [9]
5	M	49	20	Wedge resection	DD	2008, [9]
6	F	68	20	Wedge resection	AND	2008, [9]
7	M	29	28	Partial resection	AND	2008, [9]
8	F	54	30	Antrectomy gastroduodenal resection	NR	2008, [9]
9	F	58	30	Wedge resection	AND	2008, [9]
10	F	37	40	Partial resection	Recurrence, Died of other cause	2002, [9]
11	M	50	60	Tumorectomy and chemotherapy	AD	2008, [9]
12	M	42	80	Partial gastrectomy and chemothe	DD	2008, [9]
13	F	66	150	Gastrectomy and partial esophagectomy	Lost	2008, [9]
14	F	44	47	Laparoscopic wedge resection	AND	2012, [11]
15	F	38	72	Wedge resection and chemotherapy	AD	2012, [12]
16	F	42	35	Partial gastrectomy	AND	2013, [13]
17	M	22	25	Wedge resection	NR	2013, [14]
18	M	44	150	Total gastrectomy	AND	2014, [15]
19	M	62	38	Total gastrectomy and chemotherapy	AND	2014, [16]
20	F	50	80	NR	Lost	2015, [17]
21	M	36	60	NR	AD	2015, [17]
22	M	37	20	NR	NR	2015, [17]
23	M	26	NR	NR	AD	2015, [17]
24	M	58	100	NR	DD	2015, [17]
25	M	21	100	NR	DD	2015, [17]
26	M	36	50	NR	Lost	2015, [17]
27	F	54	38	NR	NR	2015, [17]
28	F	49	35	Tumorectomy	AND	2015, [18]
29	F	35	120	Tumorectomy and chemotherapy	AD	2015, [18]
30	M	56	95	resection and radiotherapy chemotherapy	AD	2016, [31]
31	F	51	9	Laparoscopy-assisted distal gastrectomy	AND	2017, [19]
32	F	27	20	Laparoscopic Gastrectomy	AND	2018, [20]
33	F	57	18	Wedge resection	NR	2018, [21]
34	M	58	63	Robotic-assisted, laparoscopic Wedge resection	AD	2019, [22]
35	M	42	30	Tumorectomy	AND	2019, [23]
36	M	54	16	Laparoscopic wedge resection	AND	2020, [25]
37	F	48	90	Distal gastrectomy and chemotherapy	NR	2020, [26]
38	M	13	110	Total gastrectomy	AND	2021, [24]
39	M	22	10	Laparoscopic partial gastrectomy	AND	2021, [27]
40	F	38	10	Resected surgically	NR	2021, [27]
41	M	72	13	Resected surgically	NR	2021, [27]
42	F	32	35	Partial gastrectomy	AND	2021, [28]
43	F	43	10	Laparoscopic intragastric resection	AND	2021, [29]
44	F	59	NR	NR	NR	2021, [30]
45	M	59	8	LECS	AND	Present case

AND, alive with no evidence of disease; AD, alive with disease; DD, died of disease, NR, not reported; LECS, laparoscopic endoscopic cooperative surgery

The 5-year survival rate of synovial sarcoma is reported to be 75% [32], and the 10-year survival rate is 34% [33]. However, patients with tumor diameters less than 5 cm have been shown to have a 10-year survival rate of 100% [33], and the poor prognostic factor was reported to be tumor diameter 5 cm or more, microscopically positive margins, 10 thread divisions or more at 10 high-power fields [33]. For primary synovial sarcoma of the stomach, Krupinska et al. found that patients with tumors larger than 72 mm had a significantly lower probability of survival and that histological subtype could influence the prognosis. In the monophasic subtype group, only one patient died (1/20), whereas in the group with biphasic or poorly differentiated tumors, the percentage of deaths was significantly higher [26].

Regarding treatment, there are reports that synovial sarcoma is characterized by less lymph node metastasis than other soft tissue sarcomas [34] and it is often locally resected considering tumor size. The indication for LECS for synovial sarcoma was considered to be patients with differentiated tumors less than 5 cm in size and no suspicion of lymph node metastasis on preoperative examination.

**Fig. 4:**
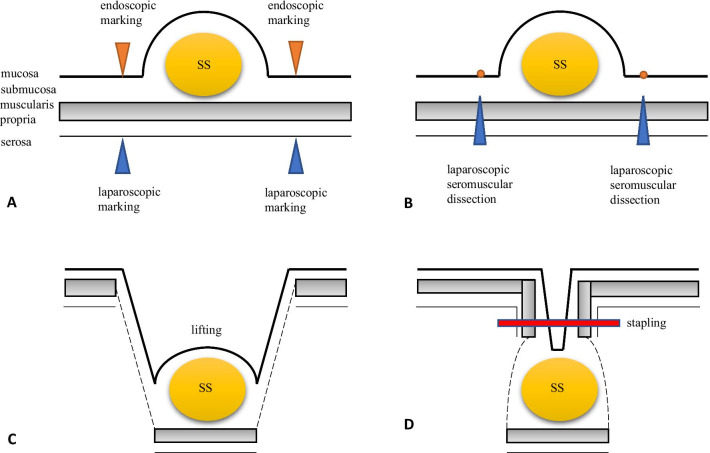
Scheme of the combination of laparoscopic and endoscopic approaches to neoplasia with a non-exposure technique (CLEAN-NET) procedure. **A** An endoscopic marking around the tumor from within the lumen of the stomach and laparoscopic marking around the tumor on the serosa contributed to the endoscopic confirmation of the resection line. **B** Laparoscopic seromuscular dissection around the tumor along the resection line. **C** The tumor was lifted toward the abdominal cavity for obtaining definite margin-free resection. **D** Full-layer resection of the stomach with a laparoscopic linear stapling device

A total of 44 cases of primary gastric synovial sarcoma have been reported thus far, and surgical procedures conducted were as follows: tumorectomy in five cases, wedge resection in nine cases, partial gastrectomy in seven cases, gastrectomy in four cases, distal gastrectomy in two cases, total gastrectomy in three cases, antrectomy in one case, intragastric resection in one case, and surgical resection in two cases. No data were available in ten cases [24, 26–30]. Thus, LECS procedure has not been reported. LECS was reported by Hiki et al. as a surgical treatment for gastrointestinal stromal tumor [35] and is now called “classical LECS [36]”. The operation time is shortened and the amount of bleeding is reduced with the minimum excision range of the gastric serosa regardless of the tumor location. The advantages of LECS over laparoscopic or robot-assisted wedge resection or partial resection are that with LECS, the resection area of the gastric serosa is smaller, thus minimizing post-resection gastric deformation, and that the resection area is sufficient for tumors of the luminal growth type that cannot be seen from the serosa [35, 36]. Inoue et al. reported CLEAN-NET as a method of local resection using an endoscope that does not require opening of the stomach wall [37]. Since the lesion in this case had a delle, and there was a possibility of dissemination of tumor cells on using classical LECS, the CLEAN-NET resection method was selected. The procedure of CLEAN-NET for SMTs is as follows (Fig. 4): (i) endoscopic marking around the tumor from within the lumen of the stomach; (ii) laparoscopic marking around the tumor on the serosa assisted by the endoscopic confirmation of the resection line; (iii) laparoscopic seromuscular dissection around the tumor along the resection line; (iv) lifting the tumor toward the abdominal cavity for obtaining definite margin-free resection; (v) full-layer resection with a laparoscopic linear stapling device; (vi) transabdominal retrieval of the specimen wrapped with a collecting bag; and (vii) checking the site by intraoperative endoscopy after resection [19]. In the present case, at the time of surgery, the presence of the tumor could not be identified from the serosal surface and consequently, the tumor could be resected using an endoscope with a sufficient margin, without exposing it. As a result, the resection margin was negative, and post-gastrectomy syndrome was not observed.

Regarding the prognosis after surgery, Kering et al. reported that the rate of distant metastasis of synovial sarcoma was 50–70%, and that local recurrence and distant metastasis in the later stage are likely to occur. They concluded that long-term follow-up for more than 10 years after surgery is necessary [38]. Therefore, careful follow-up of our patient is necessary.

As a treatment option, doxorubicin monotherapy is recommended for unresectable soft tissue sarcomas [39] and has been reported to be useful as adjuvant chemotherapy for resectable localized soft tissue sarcomas [40]. In a report of gastric synovial sarcoma, all tumors receiving adjuvant chemotherapy were larger than 3 cm, and all but one case of less than 3 cm survived without recurrence. The one patient that did not survive had poorly differentiated tumors [10]. The usefulness of radiation therapy in local control has been reported; it was used as adjuvant therapy after surgery [41].

Recently published reports of primary gastric synovial sarcoma, an extremely rare tumor, have aided in the improvement of diagnostic methods, such as molecular biological analysis. Since surgery is the only curative strategy, it is the surgeon's responsibility to ensure the resection margin and to select and perform the appropriate surgical technique to avoid dissemination of the tumor. The optimal method of surgery and selection of treatment will be determined based on the accumulation of more data from an increasing number of published cases in the future.

## Conclusions

Resection with LECS, which has not been reported before, was effective for small synovial sarcomas that could not be confirmed laparoscopically. With the CLEAN-NET procedure, it was possible to excise the tumor with the minimum excision range of the gastric serosa without opening the gastric wall.

## Supplementary Information

**Additional file 1.** The whole tumor circumference was marked on the serosa laparoscopically, and the endoscope was used as a guide around the tumor. The seromuscular layer and the submucosal layer were completely cut. The tumor covered in the mucosa was towed outside the gastric wall, and whole-layer resection was performed using a 60-mm linear stapler to confirm that the tumor was not sandwiched.

## Data Availability

The data supporting the conclusions of this study are included within the article.

## Abbreviations

CLEAN-NET: Combination of laparoscopic and endoscopic approaches to neoplasia with a non-exposure technique
EGD: Esophagogastroduodenoscopy
FISH: Fluorescence in situ hybridization
LECS: Laparoscopic endoscopic cooperative surgery
SMT: Submucosal tumor
SS: Synovial sarcoma

## References

[1] Goldblum J, Folpe A, Weiss S (2020). Enzinger & weiss's soft tissue tumors.

[2] Board WCoTE. WHO Classification of tumours, soft tissue & bone tumours, 5th ed. pp. ed2020.

[3] Tsuji S, Hisaoka M, Morimitsu Y, Hashimoto H, Shimajiri S, Komiya S (1998). Detection of SYT-SSX fusion transcripts in synovial sarcoma by reverse transcription-polymerase chain reaction using archival paraffin-embedded tissues. Am J Pathol.

[4] Saito T, Nagai M, Ladanyi M (2006). SYT-SSX1 and SYT-SSX2 interfere with repression of E-cadherin by snail and slug: a potential mechanism for aberrant mesenchymal to epithelial transition in human synovial sarcoma. Cancer Res.

[5] de Bruijn DR, Allander SV, van Dijk AH, Willemse MP, Thijssen J, van Groningen JJ (2006). The synovial-sarcoma-associated SS18-SSX2 fusion protein induces epigenetic gene (de)regulation. Cancer Res.

[6] Chuang HC, Hsu SC, Huang CG, Hsueh S, Ng KF, Chen TC (2013). Reappraisal of TLE-1 immunohistochemical staining and molecular detection of SS18-SSX fusion transcripts for synovial sarcoma. Pathol Int.

[7] Arce L, Pate KT, Waterman ML (2009). Groucho binds two conserved regions of LEF-1 for HDAC-dependent repression. BMC Cancer.

[8] Billings SD, Meisner LF, Cummings OW, Tejada E (2000). Synovial sarcoma of the upper digestive tract: a report of two cases with demonstration of the X;18 translocation by fluorescence in situ hybridization. Mod Pathol.

[9] Akhunji S, Musil I, de Leon BA, Bhattacharyya A, Cranmer LD (2007). Synovial sarcoma arising in the gastric wall: case report and literature review. Cancer Therapy.

[10] Makhlouf HR, Ahrens W, Agarwal B, Dow N, Marshalleck JJ, Lee EL (2008). Synovial sarcoma of the stomach: a clinicopathologic, immunohistochemical, and molecular genetic study of 10 cases. Am J Surg Pathol.

[11] Sinniah RP, Roche E, Cameron D (2012). GI synovial sarcomas. Clin Transl Gastroenterol.

[12] Wang CC, Wu MC, Lin MT, Lee JC (2012). Primary gastric synovial sarcoma. J Formos Med Assoc.

[13] Kamata K, Wada R, Yajima N, Sawada M, Wakasa H, Yagihashi S (2013). Primary gastric synovial sarcoma: molecular diagnosis and prediction of prognosis. Clin J Gastroenterol.

[14] Sahara S, Otsuki Y, Egawa Y, Shimizu S, Yoshizawa Y, Hosoda Y (2013). Primary synovial sarcoma of the stomach–a case report and review of the literature. Pathol Res Pract.

[15] Torres Rivas HE, Fernández S, Fresno MF (2014). Primary gastric synovial sarcoma. Pathology.

[16] Michot N, Robert PE, De Muret A, Marques F, de Calan L, Benchellal Z (2014). Gastric synovial sarcoma: case report and systematic review of literature. J Gastrointest Cancer.

[17] Romeo S, Rossi S, Acosta Marín M, Canal F, Sbaraglia M, Laurino L (2015). Primary Synovial Sarcoma (SS) of the digestive system: a molecular and clinicopathological study of fifteen cases. Clin Sarcoma Res.

[18] Wong NA, Campbell F, Shepherd NA (2015). Abdominal monophasic synovial sarcoma is a morphological and immunohistochemical mimic of gastrointestinal stromal tumour. Histopathology.

[19] So IT, Cho KB, Lee JY, Kim SJ, Jung HI, Choi JH (2017). A primary gastric synovial sarcoma: a case report and literature review. Medicine (Baltimore).

[20] Ogino S, Konishi H, Ichikawa D, Hamada J, Shoda K, Arita T (2018). Detection of fusion gene in cell-free DNA of a gastric synovial sarcoma. World J Gastroenterol.

[21] Olsen G, Beal EW, Pfeil S, Dillhoff M (2018). Primary gastric synovial sarcoma mimicking a gastrointestinal stromal tumor (GIST): gastric synovial sarcoma. J Gastrointest Surg.

[22] Hu S, Wong K, Ramesh KH, Villanueva-Siles E, Panarelli N, In H (2019). Diffuse, aggressive metastatic progression after minimally invasive local resection of primary gastric synovial sarcoma: a case report and systematic review of the literature. J Gastrointest Cancer.

[23] Fuente I, Bruballa R, Corradetti S, Cavadas D, Beskow A, Wright F (2019). Gastric synovial sarcoma. J Gastrointest Surg.

[24] Manohar A, Gopal C, Premalata CS, Kumar RV, Patil Okaly GV, Somashekhar SP (2021). Primary gastric synovial sarcoma in a child: a case report and review of the literature. J Pediatr Hematol Oncol.

[25] Wong HK, Law S, Collins R (2020). Gastric synovial sarcoma: a case report and literature review. Hong Kong Med J.

[26] Kurpińska M, Kaznowska E, Kruczak A, Mularz K, Adamczyk A, Długosz J (2020). Synovial sarcoma of the stomach: case report and systematic review of the literature. Pol J Pathol.

[27] Kuboyama Y, Yamada Y, Kohashi K, Toda Y, Kawakami K, Kitahara D (2021). Three cases of synovial sarcoma of gastric wall: a case report and review of the literature. Pathol Res Pract.

[28] Marchand Crety C, Bellefqih S, Amroun K, Garbar C, Felici F (2021). Primary gastric synovial sarcoma: a case report and literature review. Int J Surg Case Rep.

[29] Rivelli M, Fernandes E, Conti C, Bernardoni L, Pecori S, Cingarlini S (2021). Laparoscopic intragastric resection of gastric synovial sarcoma: report of the first ever case with video demonstration. World J Surg Oncol.

[30] Kinowaki Y, Abe S, Tomii S, Yukimori A, Akashi T, Tokunaga M (2021). Synovial sarcoma of the stomach: a case report and a systematic review of literature. Clin J Gastroenterol..

[31] Samuel T, Norly S, Ros'aini P (2016). Gastric ulcer that turned out to be metastasis of a synovial sarcoma: a case report and literature review. Med J Malaysia.

[32] Lewis JJ, Antonescu CR, Leung DH, Blumberg D, Healey JH, Woodruff JM (2000). Synovial sarcoma: a multivariate analysis of prognostic factors in 112 patients with primary localized tumors of the extremity. J Clin Oncol.

[33] Singer S, Baldini EH, Demetri GD, Fletcher JA, Corson JM (1996). Synovial sarcoma: prognostic significance of tumor size, margin of resection, and mitotic activity for survival. J Clin Oncol.

[34] Jacobs AJ, Morris CD, Levin AS (2018). Synovial sarcoma is not associated with a higher risk of lymph node metastasis compared with other soft tissue sarcomas. Clin Orthop Relat Res.

[35] Hiki N, Yamamoto Y, Fukunaga T, Yamaguchi T, Nunobe S, Tokunaga M (2008). Laparoscopic and endoscopic cooperative surgery for gastrointestinal stromal tumor dissection. Surg Endosc.

[36] Hiki N, Nunobe S (2019). Laparoscopic endoscopic cooperative surgery (LECS) for the gastrointestinal tract: updated indications. Ann Gastroenterol Surg.

[37] Inoue H, Ikeda H, Hosoya T, Yoshida A, Onimaru M, Suzuki M (2012). Endoscopic mucosal resection, endoscopic submucosal dissection, and beyond: full-layer resection for gastric cancer with nonexposure technique (CLEAN-NET). Surg Oncol Clin N Am.

[38] Krieg AH, Hefti F, Speth BM, Jundt G, Guillou L, Exner UG (2011). Synovial sarcomas usually metastasize after >5 years: a multicenter retrospective analysis with minimum follow-up of 10 years for survivors. Ann Oncol.

[39] Bramwell VH, Anderson D, Charette ML, Group SDS (2003). Doxorubicin-based chemotherapy for the palliative treatment of adult patients with locally advanced or metastatic soft tissue sarcoma. Cochrane Database Syst Rev.

[40] Pervaiz N, Colterjohn N, Farrokhyar F, Tozer R, Figueredo A, Ghert M (2008). A systematic meta-analysis of randomized controlled trials of adjuvant chemotherapy for localized resectable soft-tissue sarcoma. Cancer.

[41] Rosenberg SA, Tepper J, Glatstein E, Costa J, Baker A, Brennan M (1982). The treatment of soft-tissue sarcomas of the extremities: prospective randomized evaluations of (1) limb-sparing surgery plus radiation therapy compared with amputation and (2) the role of adjuvant chemotherapy. Ann Surg.

